# Meeting of the Central New York Homœopathic Medical Society

**Published:** 1885-08

**Authors:** E. B. Nash

**Affiliations:** Secretary


### MEETING OF THE CENTRAL NEW YORK HOMŒOPATHIC MEDICAL SOCIETY,

### HELD MARCH 19TH, 1885, AT THE OFFICE OF DR. HAWLEY, SYRACUSE, N. Y.

The meeting was called to order by the Vice-President, Dr. Young, at 10 A. M.

Members present—Drs. Hawley, Boyce, Young, Seward, and Carr.

Dr. Hawley nominated Dr. Carr Secretary *pro tem.* Elected.

No report from Committee on Credentials.

Secretary's and Treasurer's report not received, owing to their absence.

The President (Dr. Stowe) having arrived, Dr. Young vacated the chair.

The following Committee on Credentials was appointed *pro tem.:* Drs. Boyce, Young, and Seward.

Communication of the President received with the remarks that in New England pneumonia has been prevalent. Allopathy lost seventy-five per cent.; Homœopathy much less, as usual.

Dr. Seward reported a case of typhoid called to mind by Dr. Gregg's paper (published in HOMŒOPATHIC PHYSICIAN) on the too frequent repetition of the remedy.

Drs. Brewster and Harris arrived.

Dr. Boyce read from Hahnemann's *Organon* on repetition of the dose.

Dr. Wells arrived.

Dr. Boyce read a paper on Hahnemann's three precautions.

Dr. Hawley moved that the paper be forwarded to THE HOMŒOPATHIC PHYSICIAN for publication. Carried.

Dr. Hawley reported a case of secondary syphilis requiring repeated doses of Nit. ac.$^{5m}$

Dr. Brewster—Are we to conclude that in some cases we must repeat the doses?

Dr. Hawley answered affirmatively.

Dr. Brewster reports a case of renal colic relieved in previous attacks by Morphine, which now failed. Bell. relieved, and the nausea from the Morphine was relieved by Nux vom.

Meeting adjourned till 1.30 P. M.

Called to order at 1.45 P. M.; President in the chair.

Dr. Boyce introduced the subject of the meeting of the I. H. A. to be held here in June.

After some discussion, Dr. Wells moved "That a committee of three (with Dr. Hawley as chairman) be appointed to confer with the I. H. A. in relation to the meeting in June and to make all necessary arrangements for the same." Carried.

Chair appointed Drs. Hawley, Seward, and Brewster.

Drs. Hawley, Wallace, and Stowe each presented cases for advice.

The I. H. A. holds its meetings on the 23d, 24th, and 25th of June at the Court-house.

Adjourned to third Thursday in June, at Dr. Hawley's office.

E. B. NASH, Secretary.

